# Glutathione and proline can coordinately make plants withstand the joint attack of metal(loid) and salinity stresses

**DOI:** 10.3389/fpls.2014.00662

**Published:** 2014-11-21

**Authors:** Naser A. Anjum, Ibrahim M. Aref, Armando C. Duarte, Eduarda Pereira, Iqbal Ahmad, Muhammad Iqbal

**Affiliations:** ^1^Department of Botany, Faculty of Science, Hamdard UniversityNew Delhi, India; ^2^CESAM-Centre for Environmental and Marine Studies and Department of Chemistry, University of AveiroAveiro, Portugal; ^3^Plant Production Department, College of Food and Agricultural Sciences, King Saud UniversityRiyadh, Saudi Arabia

**Keywords:** glutathione, metal/metalloids, osmotic stress, oxidative stress, proline, redox homeostasis, salinity stress

## Introduction

Agricultural soils in the vicinity of extensive anthropogenic activities may exhibit salinity together with high levels of metals/metalloids (hereafter termed as “metal/s”) as co-stressors. Elevated concentrations of metals (such as As, Cd, Cr, Hg, Ni, and Pb) may affect photosynthetic apparatus, electron transport chain and chlorophyll biosynthesis, induce cellular damage, impair cellular redox homeostasis, and finally cause cellular metabolic arrest (Anjum et al., 2010, 2012a; Gill and Tuteja, 2010; Talukdar, 2012; Talukdar and Talukdar, 2014). Saline soil conditions, on the other hand, can cause osmotic stress that in turn can inhibit cell expansion and cell division, impact stomatal closure, induce cell turgor *via* lowering water potential, and alter the normal homeostasis of cells (Miller et al., 2010). However, the generation of osmotic stress through impaired plant water relations, and oxidative stress caused by uncontrolled generation of varied reactive oxygen species (ROS; such as such as -OH, H_2_O_2_, O^−^_2_) are common in plants exposed to high levels of salinity and/or metals (Benavides et al., 2005; Anjum et al., 2010, 2012a).

**Figure 1:**
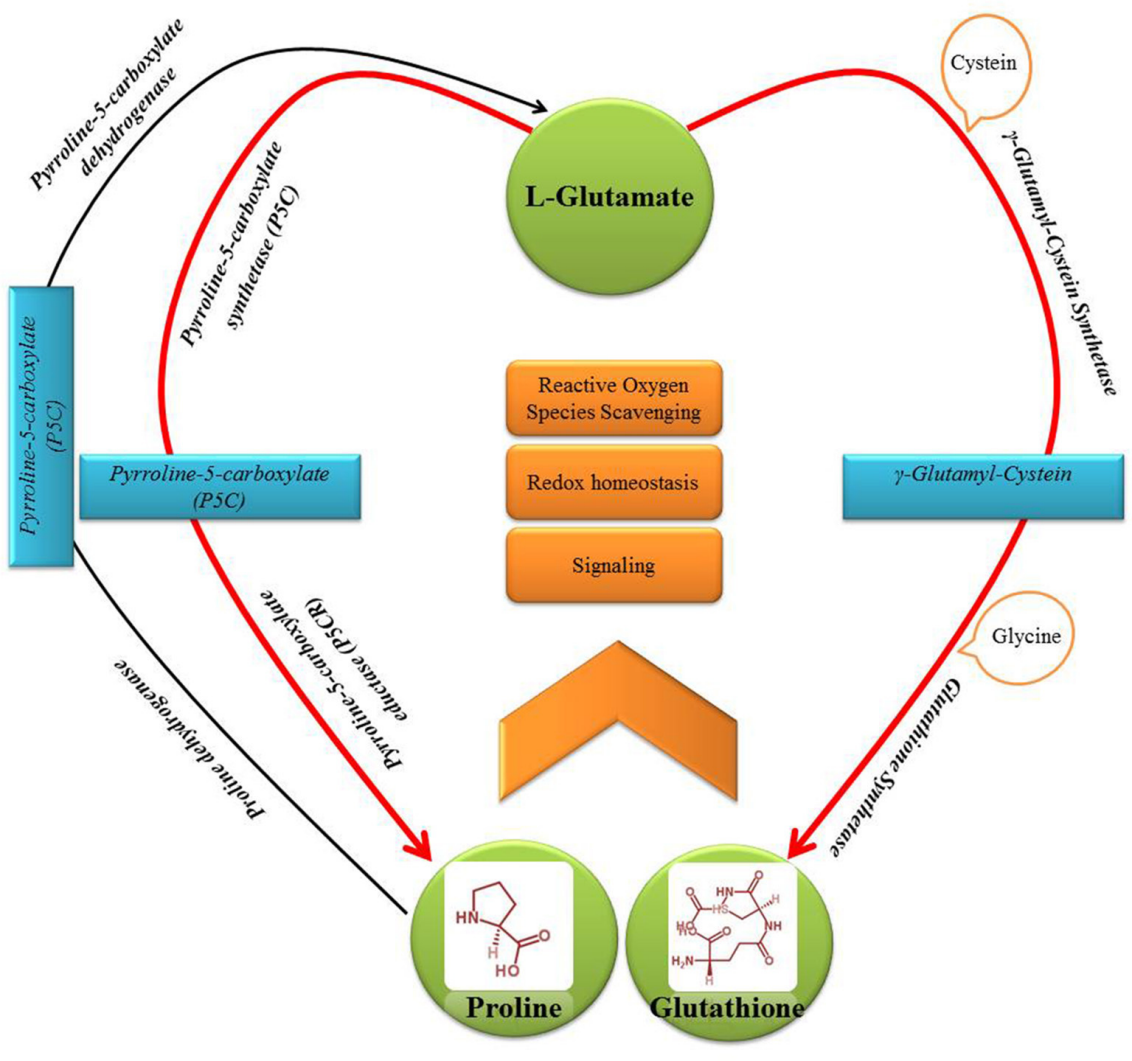
**Schematic representation of the points of interrelationships in the major metabolic pathway of sulfur-rich peptide—glutathione (GSH) and nitrogenous and proteogenic amino acid—proline (Pro)**.

Diverse plant taxa have been reported to adapt metabolically to salinity and exposure to metals by enhancing synthesis of sulfur (S)-rich peptides (such as glutathione, GSH) and low-molecular-weight nitrogenous and proteogenic amino acids/osmolytes (such as proline, Pro) (Khan et al., 2009; Anjum et al., 2010, 2012a; Talukdar, 2012; Kishor and Sreenivasulu, 2014; Talukdar and Talukdar, 2014). Nevertheless, both GSH and Pro share L-glutamate as a common biosynthesis precursor (Moat et al., 2003) (Figure 1). However, very little or no effort has been made so far to dissect the intricacies of potential metabolic interrelationships between the GSH and Pro induction either under salinity/osmotic or metal stress conditions.

Therefore, we discuss and interpret through this note the facts related with the mainstays (chemistry, biosynthesis, compartmentalization, significance) commonly and potentially shared by these two enigmatic compounds (GSH and Pro) in plants. The outcome of the present endeavor can be useful in designing future research aimed at sustainably alleviating isolated and/or joint impact of metal and salinity stresses in crop plants through exploiting the GSH and Pro metabolism.

## Cross-talks and perspectives

Both GSH and Pro, with molecular formula C_10_ H_17_ N_3_ O_6_ S and C_5_ H_9_ NO_2_, respectively, belong to the “glutamate or α-ketoglutarate” family and originate from a common precursor L-glutamate (Moat et al., 2003). Although cellular compartments and changing growth conditions may influence their levels, biosynthesis of both GSH (Preuss et al., 2014) and Pro (Lehmann et al., 2010) is predominantly plastidic. Of the two major GSH-biosynthesis enzymes, glutamate cysteine ligase (GCL; γ-glutamylcysteine synthetase; E.C. 6.3.2.2) is localized to plastid stroma; whereas GSH synthetase (GS; E.C. 6.3.2.3) is targeted to plastid stroma and cytosol (Ravilious and Jez, 2012). On the other hand, the Pro-biosynthesis enzymes, namely Δ1-pyrroline-5-carboxylate synthetase (P5CS) and Δ1-pyrroline-5-carboxylate reductase (P5CR), occur in cytosol and plastids (reviewed by Szabados and Savouré, 2010). Since plastids are among the major organelles with: (a) a highly oxidizing metabolic activity; (b) an intense rate of electron flow; and (c) plastid signal-mediated regulation of different cellular processes (Barajas-López et al., 2013), localization of both GSH and Pro is apt to their role as the major ROS-scavenger and singlet-oxygen quencher during photosynthesis (Szekely et al., 2008).

GSH and Pro may occur in the concentrations of few mM (2–3 mM) in various plant tissues (Noctor et al., 2002; Kishor et al., 2005). The GSH and Pro levels of plant tissues are indicators of the S (reduced) (Hubberten et al., 2012) and nitrogen (N) (Sánchez et al., 2001) nutritional status of the plant respectively. GSH and Pro have also been reported to act as sources of (reduced)-S (Anjum et al., 2010) and N (reviewed by Kishor and Sreenivasulu, 2014), respectively, under stress conditions. Additionally, their status may presumably be improved through enhancing L-glutamate level *via* N and S nutrition, respectively (Anjum et al., 2012b). Moreover, modulation of biosynthesis of GSH (Bartoli et al., 2009) and Pro (Abraham et al., 2003) is reportedly light dependent. In particular, GSH levels may depend on growth and photosynthetically active photon flux density at low light intensities (up to ca. 100 μmol m^−2^ s^−1^) (Ogawa et al., 2004). GSH (Son et al., 2014) and Pro (Sivakumar et al., 2001) can negatively/positively modulate the photosynthesis functions by influencing the activity of ribulose-1,5-bisphosphate oxygenase, an enzyme involved in the first major step of carbon fixation. Moreover, an increased intracellular ROS-availability can shift the reduced GSH toward a more oxidized GSH (i.e., GSSG) status (Anjum et al., 2010, 2012a; Noctor et al., 2012). In contrast, increased status of cellular H_2_O_2_ (or exogenous H_2_O_2_) can increase Pro level by modulating the *ex-novo* synthesis of Pro (Matysik et al., 2002). Oxidation of Pro generates NADP/NADPH cycling or redox balance (Kishor et al., 2005) that in turn may regulate the reduction of GSSG to GSH *via* GSH reductase (Anjum et al., 2010, 2012a; Noctor et al., 2012). Interaction of Pro (Iqbal et al., 2014) and GSH (Mhamdi et al., 2010; Ghanta et al., 2014) with a number of defense-related phytohormones (such as ethylene, jasmonic acid and salicylic acid) and/or their analogs has also been reported to modulate plant stress tolerance.

Both GSH (Ogawa, 2005) and Pro (Lehmann et al., 2010) perform multiple functions in plants including the modulation of plant growth and developmental processes. In particular, under metal stress, apart from the induction of GSH-based defense system (Anjum et al., 2010, 2012a; Noctor et al., 2012; Talukdar, 2012; Talukdar and Talukdar, 2014), elevated accumulation of osmolytes such as Pro has been extensively noticed (reviewed by Gill et al., 2014). Under salinity stress also, in addition to the accumulation of Pro that maintains both cell turgor and cellular redox homeostasis (Lehmann et al., 2010; Szabados and Savouré, 2010; Kishor and Sreenivasulu, 2014), GSH-based defense system is activated to maintain reduced cellular redox environment *via* metabolizing the varied ROS and their reaction products (Ruiz and Blumwald, 2002; Kocsy et al., 2004). Nevertheless, reports are available on the efficient Pro-metal, GSH-metal or Pro-GSH-metal sequestration, scavenging of ROS-types and also on the maintenance of reduced cellular redox environment by GSH (Anjum et al., 2010, 2012a; Noctor et al., 2012; Talukdar, 2012; Talukdar and Talukdar, 2014) and Pro (Matysik et al., 2002; Siripornadulsil et al., 2002; Lehmann et al., 2010; Szabados and Savouré, 2010; Kishor and Sreenivasulu, 2014).

A differential coordination of other components of ascorbate (AsA)-GSH pathway (enzymes such as ascorbate peroxidase, GSH reductase, GSH peroxidase, GSH sulfo-transferase, monodehydroascorbate reductase, dehydroascorbate reductase and catalase; and non-enzymes such as AsA) with GSH (Khan et al., 2009; Anjum et al., 2012a, 2014; Talukdar, 2012; Talukdar and Talukdar, 2014) and Pro (Omidi, 2010; Hossain et al., 2011; Anjum et al., 2014; Hasanuzzaman et al., 2014) was also reported to control plant tolerance to abiotic stress factors including the metal and salinity stress. Nevertheless, the status and responses of GSH and Pro together have been little explored in the same plant under similar stress conditions (Siripornadulsil et al., 2002; Hossain et al., 2011; Anjum et al., 2014; Hasanuzzaman et al., 2014). Notably, these studies helped to infer that there exists a close relation between GSH and Pro, and that exogenous and/or synthesized/ stress-caused elevated Pro can protect plants against the metal and salinity-stress impacts by safe-guarding the activity of previous enzymatic components, improving the cellular redox environment *via* decreasing H_2_O_2_ level and maintaining an increased level of reduced GSH and GSSG/GSH ratio.

Though an increased cellular GSH status is indicative of a plant's capacity to tolerate different stress pressures (Khan et al., 2009; Anjum et al., 2010, 2012a; Talukdar, 2012; Noctor et al., 2012; Talukdar and Talukdar, 2014), it is debatable whether accumulation of Pro is a plant response to abiotic stresses or it is associated with stress tolerance (Sorkheh et al., 2012; Kishor and Sreenivasulu, 2014). Also, elevated GSH is not always correlated with enhanced tolerance to stresses such as metals (Xiang et al., 2001; reviewed by Anjum et al., 2012a). Despite previous facts, as versatile redox buffers, Pro (Kishor and Sreenivasulu, 2014) and GSH (Anjum et al., 2010, 2012a; Noctor et al., 2012) have been extensively evidenced to protect cellular metabolism against a range of abiotic stresses.

The causal relationships of Pro accumulation and significance of GSH metabolism with enhanced tolerance to single stress factor (either metal or salinity) have been reported extensively in separate studies using natural variants, mutants or transgenic plants (Matysik et al., 2002; Anjum et al., 2010, 2012a; Noctor et al., 2012; Kishor and Sreenivasulu, 2014). However, significance of the potential “metabolic interrelationships” between GSH and Pro with reference to the plant's adaptive responses to prevailing multiple stressors has not been fully appreciated and the molecular insights of these relationships have yet to be developed.

Nevertheless, owing to the facts that: (a) deficiency of S and N has become extensive in agricultural soils on the globe (reviewed by Anjum et al., 2012b); (b) plant's S requirement and S metabolism are closely related to N nutrition, and the N metabolism is strongly affected by the plant's S status (Fazili et al., 2008; Anjum et al., 2012b); and (c) both GSH (Kopriva and Rennenberg, 2004; Anjum et al., 2012b) and Pro (Sánchez et al., 2001; Rais et al., 2013) are closely related to these nutrients, integrated efforts should be made to work-out the coordinated role of S and N in the GSH and Pro metabolic pathways, develop more insights into their biochemistry/physiology and molecular biology and understand potential interrelationships among different components of these pathways.

### Conflict of interest statement

The authors declare that the research was conducted in the absence of any commercial or financial relationships that could be construed as a potential conflict of interest.

## References

[B1] Abraham E. Rigo G. Szekely G. Nagy R. Koncz C. Szabados L. (2003). Light-dependent induction of proline biosynthesis by abscisic acid and salt stress is inhibited by brassinosteroid in *Arabidopsis*. Plant Mol. Biol. 51, 363–372. 10.1023/A:1022043000516 12602867

[B2] Anjum N. A. Ahmad I. Mohmood I. Pacheco M. Duarte A. C. Pereira E. (2012a). Modulation of glutathione and its related enzymes in plants' responses to toxic metals and metalloids—a review. Environ. Exp. Bot. 75, 307–324 10.1016/j.envexpbot.2011.07.002

[B3] Anjum N. A. Gill S. S. Umar S. Ahmad I. Duarte A. C. Pereira E. (2012b). Improving growth and productivity of oleiferous *Brassicas* under changing environment: significance of nitrogen and sulphur nutrition, and underlying mechanisms. Sci. World J. 2012:657808. 10.1100/2012/657808 22629181 PMC3353521

[B4] Anjum N. A. Israr M. Duarte A. C. Pereira M. E. Ahmad I. (2014). Halimione portulacoides (L.) physiological/biochemical characterization for its adaptive responses to environmental mercury exposure. Environ. Res. 131, 39–49. 10.1016/j.envres.2014.02.008 24641832

[B5] Anjum N. A. Umar S. Chan M. T. (2010). Ascorbate-Glutathione Pathway and Stress Tolerance in Plants. Dordrecht: Springer.

[B6] Barajas-López J. D. Blanco N. E. Strand Å. (2013). Plastid-to-nucleus communication, signals controlling the running of the plant cell. Biochim. Biophys. Acta Mol. Cell Res. 1833, 425–437. 10.1016/j.bbamcr.2012.06.020 22749883

[B7] Bartoli C. G. Tambussi E. A. Diego F. Foyer C. H. (2009). Control of ascorbic acid synthesis and accumulation and glutathione by the incident light red/far red ratio in *Phaseolus vulgaris* leaves. FEBS Lett. 583, 118–122. 10.1016/j.febslet.2008.11.034 19059408

[B8] Benavides M. P. Gallego S. M. Tomaro M. L. (2005). Cadmium toxicity in plants. Braz. J. Plant Physiol. 17, 21–34 10.1590/S1677-04202005000100003

[B9] Fazili I. S. Jamal A. Ahmad S. Masoodi M. Khan J. S. Abdin M. Z. (2008). Interactive effect of sulfur and nitrogen on nitrogen accumulation and harvest in oilseed crops differing in nitrogen assimilation potential. J. Plant Nutr. 31, 1203–1220 10.1080/01904160802134905

[B10] Ghanta S. Datta R. Bhattacharyya D. Sinha R. Kumar D. Hazra S.. (2014). Multistep involvement of glutathione with salicylic acid and ethylene to combat environmental stress. J. Plant Physiol. 171, 940–950. 10.1016/j.jplph.2014.03.002 24913051

[B11] Gill S. S. Gill R. Anjum N. A. (2014). Target osmoprotectants for abiotic stress tolerance in crop plants—glycine betaine and proline, in Plant Adaptation to Environmental Change: Significance of Amino Acids and Their Derivatives, eds Anjum N. A. Gill S. S. Gill R. (Wallingford, CT: CAB International), 97–108.

[B12] Gill S. S. Tuteja N. (2010). Reactive oxygen species and antioxidant machinery in abiotic stress tolerance in crop plants. Plant Physiol. Biochem. 48, 909–930. 10.1016/j.plaphy.2010.08.016 20870416

[B13] Hasanuzzaman M. Alam M. M. Rahman A. Hasanuzzaman M. Nahar K. Fujita M. (2014). Exogenous proline and glycine betaine mediated upregulation of antioxidant defense and glyoxalase systems provides better protection against salt-induced oxidative stress in two rice (*Oryza sativa* L.) varieties. Biomed. Res. Intl. 2014:757219 10.1155/2014/757219 24991566 PMC4065706

[B14] Hossain M. Hasanuzzaman M. Fujita M. (2011). Coordinate induction of antioxidant defense and glyoxalase system by exogenous proline and glycinebetaine is correlated with salt tolerance in mung bean. Front. Agric. China 5, 1–14 10.1007/s11703-010-1070-2

[B15] Hubberten H. M. Drozd A. Tran B. V. Hesse H. Hoefgen R. (2012). Local and systemic regulation of sulfur homeostasis in roots of *Arabidopsis thaliana*. Plant J. 72, 625–635. 10.1111/j.1365-313X.2012.05105.x 22775482

[B16] Iqbal N. Umar S. Khan N. A. Khan M. I. R. (2014). A new perspective of phytohormones in salinity tolerance: regulation of proline metabolism. Environ. Exp. Bot. 100, 34–42 10.1016/j.envexpbot.2013.12.006

[B17] Khan I. Ahmad A. Iqbal M. (2009). Modulation of antioxidant defence system for arsenic detoxification in Indian mustard. Ecotoxicol. Environ. Saf. 72, 626–634. 10.1016/j.ecoenv.2007.11.016 18262648

[B18] Kishor P. B. K. Sangam S. Amrutha R. N. Laxmi P. S. Naidu K. R. Rao K. R. S. S. (2005). Regulation of proline biosynthesis, degradation, uptake and transport in higher plants: its implications in plant growth and abiotic stress tolerance. Curr. Sci. 88, 424–438.

[B19] Kishor P. B. K. Sreenivasulu N. (2014). Is proline accumulation *per se* correlated with stress tolerance or is proline homeostasis a more critical issue? Plant Cell Environ. 37, 300–311. 10.1111/pce.12157 23790054

[B20] Kocsy G. Szalai G. Galiba G. (2004). Effect of osmotic stress on glutathione and hydroxymethylglutathione accumulation in wheat. J. Plant Physiol. 161, 785–794. 10.1016/j.jplph.2003.12.006 15310067

[B21] Kopriva S. Rennenberg H. (2004). Control of sulphate assimilation and glutathione synthesis: interaction with N and C metabolism. J. Exp. Bot. 55, 1831–1842. 10.1093/jxb/erh203 15286142

[B22] Lehmann S. Funck D. Szabados L. Rentsch D. (2010). Proline metabolism and transport in plant development. Amino Acids 39, 949–962. 10.1007/s00726-010-0525-3 20204435

[B23] Matysik J. Alia Bhalu B. Mohanty P. (2002). Molecular mechanisms of quenching of reactive oxygen species by proline under stress in plants. Curr. Sci. 82, 525–532.

[B24] Mhamdi A. Hager J. Chaouch S. Queval G. Han Y. Taconnat L.. (2010). *Arabidopsis* GLUTATHIONE REDUCTASE 1 plays a crucial role in leaf responses to intracellular H_2_O_2_ and in ensuring appropriate gene expression through both salicylic acid and jasmonic acid signaling pathways. Plant Physiol. 153, 1144–1160. 10.1104/pp.110.153767 20488891 PMC2899936

[B25] Miller G. Suzuki N. Ciftci-Yilmaz S. Mittler R. (2010). Reactive oxygen species homeostasis and signalling during drought and salinity stresses. Plant Cell Environ. 33, 453–467. 10.1111/j.1365-3040.2009.02041.x 19712065

[B26] Moat A. G. Foster J. W. Spector M. P. (2003). Biosynthesis and metabolism of amino acids, in Microbial Physiology, eds Moat A. G. Foster J. W. Spector M. P. (New York, NY: John Wiley & Sons), 503–544.

[B27] Noctor G. Gomez L. A. Vanacker H. Foyer C. H. (2002). Interactions between biosynthesis, comparmentation and transport in the control of glutathione homeostasis and signaling. J. Exp. Bot. 53, 1283–1304. 10.1093/jexbot/53.372.1283 11997376

[B28] Noctor G. Mhamdi A. Chaouch S. Han Y. I. Neukermans J. Marquez−Garcia B. E. L. E. N.. (2012). Glutathione in plants: an integrated overview. Plant Cell Environ. 35, 454–484. 10.1111/j.1365-3040.2011.02400.x 21777251

[B29] Ogawa K. (2005). Glutathione-associated regulation of plant growth and stress responses. Antioxid. Redox Signal 7, 973–981. 10.1089/ars.2005.7.973 15998252

[B30] Ogawa K. Hatano-Iwasaki A. Yanagida M. Iwabuchi M. (2004). Level of glutathione is regulated by ATP-dependent ligation of glutamate and cysteine through photosynthesis in *Arabidopsis thaliana*: mechanism of strong interaction of light intensity with flowering. Plant Cell Physiol. 45, 1–8. 10.1093/pcp/pch008 14749480

[B31] Omidi H. (2010). Changes of proline content and activity of antioxidative enzymes in two canola genotype under drought stress. Amer. J. Plant Physiol. 5, 338–349 10.3923/ajpp.2010.338.349

[B32] Preuss M. L. Cameron J. C. Berg R. H. Jez J. M. (2014). Immunolocalization of glutathione biosynthesis enzymes in *Arabidopsis thaliana*. Plant Physiol. Biochem. 75, 9–13. 10.1016/j.plaphy.2013.11.027 24361505

[B33] Rais L. Masood A. Inam A. Khan N. (2013). Sulfur and nitrogen co-ordinately improve photosynthetic efficiency, growth and proline accumulation in two cultivars of mustard under salt stress. J. Plant Biochem. Physiol. 1:101 10.4172/jpbp.1000101

[B34] Ravilious G. E. Jez J. M. (2012). Stuctural biology of plant sulfur metabolism: from assimilation to biosynthesis. Nat. Prod. Rep. 29, 1138–1152. 10.1039/c2np20009k 22610545

[B35] Ruiz J. Blumwald E. (2002). Salinity-induced glutathione synthesis in *Brassica napus*. Planta 214, 965–969. 10.1007/s00425-002-0748-y 11941474

[B36] Sánchez E. López-Lefebre L. R. García P. C. Rivero R. M. Ruiz J. M. Romero L. (2001). Proline metabolism in response to highest nitrogen dosages in green bean plants (*Phaseolus vulgaris* L. cv. Strike). J. Plant Physiol. 158, 593–598 10.1078/0176-1617-00268

[B37] Siripornadulsil S. Traina S. Verma D. P. Sayre R. T. (2002). Molecular mechanisms of proline-mediated tolerance to toxic heavy metals in transgenic microalgae. Plant Cell 14, 2837–2847. 10.1105/tpc.004853 12417705 PMC152731

[B38] Sivakumar P. Sharmila P. Saradhi P. P. (2001). Proline suppresses rubisco activity by dissociating small subunits from holoenzyme. Biochem. Biophys. Res. Commun. 282, 236–241. 10.1006/bbrc.2001.4540 11263997

[B39] Son J. A. Narayanankutty D. P. Roh K. S. (2014). Influence of exogenous application of glutathione on rubisco and rubisco activase in heavy metal-stressed tobacco plant grown *in vitro*. Saudi J. Biol. Sci. 21, 89–97. 10.1016/j.sjbs.2013.06.002 24596504 PMC3937471

[B40] Sorkheh K. Shiran B. Khodambashi M. Rouhi V. Mosavei S. Sofo A. (2012). Exogenous proline alleviates the effects of H_2_O_2_-induced oxidative stress in wild almond species. Russ. J. Plant Physiol. 59, 788–798. 10.1134/S102144371206016717188658

[B41] Szabados L. Savouré A. (2010). Proline: a multifunctional amino acid. Trend Plant Sci. 15, 89–97. 10.1016/j.tplants.2009.11.009 20036181

[B42] Szekely G. Abraham E. Cseplo A. Rigo G. Zsigmond L. Csiszár J.. (2008). Duplicated P5CS genes of *Arabidopsis* play distinct roles in stress regulation and developmental control of proline biosynthesis. Plant J. 53, 11–28. 10.1111/j.1365-313X.2007.03318.x 17971042

[B43] Talukdar D. (2012). An induced glutathione-deficient mutant in grass pea (*Lathyrus sativus* L.): modifications in plant morphology, alteration in antioxidant activities and increased sensitivity to cadmium. Biorem. Biodiv. Bioavail. 6, 75–86.

[B44] Talukdar D. Talukdar T. (2014). Coordinated response of sulfate transport, cysteine biosynthesis, and glutathione-mediated antioxidant defense in lentil (*Lens culinaris* Medik.) genotypes exposed to arsenic. Protoplasma 251, 839–855. 10.1007/s00709-013-0586-8 24276371

[B45] Xiang C. Werner B. L. Christensen E. M. Oliver D. J. (2001). The biological functions of glutathione revisited in *Arabidopsis* transgenic plants with altered glutathione levels. Plant Physiol. 126, 564–574. 10.1104/pp.126.2.564 11402187 PMC111149

